# Drug–Drug Interactions of Hydroxychloroquine and Chloroquine in Older Patients with COVID-19 during the First Pandemic Waves: The GeroCovid Observational Study

**DOI:** 10.3390/reports7020042

**Published:** 2024-05-23

**Authors:** Caterina Trevisan, Andrea Cignarella, Andrea Grandieri, Giuseppe Sergi, Stefano Fumagalli, Fabio Monzani, Chukwuma Okoye, Giuseppe Bellelli, Alba Malara, Pietro Gareri, Stefano Volpato, Raffaele Antonelli Incalzi, The GeroCovid Acute Ward Working Group

**Affiliations:** 1 Geriatric and Orthogeriatric Unit, Department of Medical Science, University of Ferrara, 44124 Ferrara, Italy; andrea.grandieri30@gmail.com (A.G.); stefano.volpato@unife.it (S.V.); 2 Department of Medicine (DIMED), University of Padua, 35121 Padua, Italy; andrea.cignarella@unipd.it (A.C.); giuseppe.sergi@unipd.it (G.S.); 3 Department of Biomedicine and Prevention, University of Rome “Tor Vergata”, 00133 Rome, Italy; 4 Department of Experimental and Clinical Medicine, University of Florence and Division of Geriatric and Intensive Care Medicine, Azienda Ospedaliero-Universitaria Careggi, 50134 Florence, Italy; stefano.fumagalli@unifi.it; 5 Geriatrics Unit, Department of Clinical and Experimental Medicine, University of Pisa, 56126 Pisa, Italy; fabio.monzani@unipi.it (F.M.); chukwuma.okoye@unimib.it (C.O.); 6 School of Medicine and Surgery, University of Milano-Bicocca and Acute Geriatric Unit, Fondazione IRCCS San Gerardo dei Tintori, 20900 Monza, Italy; giuseppe.bellelli@unimib.it; 7 ANASTE-Humanitas Foundation, 00192 Rome, Italy; albamalara@gmail.com; 8 Center for Cognitive Disorders and Dementia (CDCD) Catanzaro Lido, ASP Catanzaro, 88100 Catanzaro, Italy; pietro.gareri@gmail.com; 9 Unit of Geriatrics, Department of Medicine, Campus Bio-Medico University, 00128 Rome, Italy; r.antonelli@policlinicocampus.it

**Keywords:** hydroxychloroquine, chloroquine, COVID-19, drug interactions

## Abstract

Objective: Chloroquine (CQ) and hydroxychloroquine (HCQ) were used as off-label treatments for SARS-CoV-2 infection during the first pandemic waves. The urgency of combatting COVID-19 led to the dissemination of medical recommendations with a scarce awareness of possible drug–drug interactions. This issue primarily concerned people already taking multiple medications, such as older individuals. We estimated the prevalence of drug interactions with CQ or HCQ in COVID-19 inpatients during the first pandemic waves and their possible association with hospitalization-related outcomes. Methods: This study considers 487 patients aged ≥60, hospitalized for COVID-19 from March to December 2020, and treated with CQ or HCQ. Data on acute and chronic therapies and hospitalization length and outcomes were derived from medical records. The presence of drugs potentially interacting with CQ and HCQ was identified based on published literature and drug databases. Results: In our sample (mean age 77.1 years, 47.8% females), 255 (52.4%) patients presented with one drug interaction with CQ or HCQ, and 114 (23.4%) had more than two interactions. The most frequent drugs potentially interacting with CQ or HCQ were lopinavir/ritonavir (50.4%), azithromycin (47.2%), tocilizumab (15.4%), levofloxacin (8.7%), clarithromycin (6.0%), amlodipine (3.3%), and trazodone (2.4%). No substantial differences in the duration and outcomes of the hospitalization emerged as a function of the presence of drug–drug interactions. Conclusions: Many older patients prescribed with CQ or HCQ, which have lately proved ineffective against COVID-19, were exposed to the risk of drug–drug interaction. This underlines that medical recommendations should undergo careful peer review before being widely disseminated, even in emergencies like a pandemic.

## 1. Introduction

Since the beginning of 2020, the spread of the COVID-19 pandemic raised extreme challenges for healthcare professionals. Among these, patient overload and the scarcity of resources and specific therapies resulted in a high burden even for the most advanced health systems. In this context, the urgency of identifying a valid medication against SARS-CoV-2 infection and the limited scientific knowledge on the topic led to the dissemination of various non-evidence-based medical recommendations, including the use of off-label medications, such as chloroquine (CQ) and hydroxychloroquine (HCQ) [1].

In clinical practice, both CQ and HCQ were initially prescribed for malaria treatment and prevention but, nowadays, are used for autoimmune diseases. The indication of using these medications for SARS-CoV-2 infection was supported by studies showing that CQ in vitro was active against the SARS coronavirus [1]. Subsequently, during the first pandemic wave, the awareness of the potential beneficial effects of CQ and HCQ on patients with COVID-19 arose from several interventional and observational clinical trials that, however, had significant methodological limitations [2]. Evidence for the efficacy and tolerability profile of CQ and HCQ treatment in COVID-19 patients was ultimately limited, and after a few months, these treatments were found ineffective for such an infection [3].

These facts should be carefully reanalyzed from a constructive view that, even after a few years, can help the scientific and medical communities improve in working in synergy and develop solid recommendations for these complex scenarios. For instance, the potential detrimental consequences of introducing a new treatment in patients with multiple ongoing medications, such as the oldest ones, are worthy of consideration. As is well-known, age-related multimorbidity frequently accounts for polypharmacological regimens [4]. Patients taking several drugs are, therefore, more prone to adverse drug reactions and drug–drug interactions [5,6]. In the latter cases, in particular, two or more drugs interact with each other, and such an interaction can affect the medications’ effectiveness or toxicity [7].

HCQ/CQ are extensively tissue-bound, particularly to melanin-containing tissue such as the retina, and have long and variable plasma elimination half-lives (approximately 50 days) because of a high distribution volume. This long residence time predisposes the drugs to interactions, which may be mediated by both pharmacokinetic (e.g., CYP2D6 inhibition) and pharmacodynamic mechanisms (e.g., enhancing the hypoglycemic and/or QTc-prolonging effect of other agents) [8]. For example, HCQ/CQ use has been found to increase metoprolol or cyclosporin A exposure [9,10].

Despite the attention to these issues in routine practice and the warnings raised by some authors [6,11,12,13,14,15,16], the possible interactions between newly prescribed and ongoing medications during the pandemic were only scarcely considered due to the need to prioritize fighting the disease [9,10]. Therefore, the aims of the present study were (1) to estimate the prevalence of potential drug–drug interactions associated with the use of CQ or HCQ in COVID-19 patients aged over 60 years hospitalized during the first and second pandemic waves; and (2) to analyze if these interactions may have influenced patients’ hospital course and clinical outcomes.

## 2. Methods

### 2.1. Study Population

This study analyzes data from the GeroCovid Observational study—acute ward cohort, a multicenter initiative promoted by the Italian Society of Gerontology and Geriatrics that involved individuals aged ≥60 years hospitalized for SARS-CoV-2 infection in Italy and Norway. Details on this initiative can be found in previous publications [17,18]. Patients were observed retrospectively and/or prospectively between March and December 2020, and sociodemographic and health-related data were recorded in an electronic registry developed by Bluecompanion Ltd. (London, UK). The GeroCovid Observational study protocol, composed of different cohorts, was registered in ClinicalTrials.gov (NCT04379440). The study protocol was approved by the Ethical Committee of each involved center, and the participants gave informed consent for taking part in the study.

For the present study, from the initial sample of 1276 participants, we excluded individuals with missing data on chronically used medications (n = 281) and age (n = 6) and those who were not treated with CQ or HCQ during their hospitalization (n = 502). We obtained a final analytical sample of 487 inpatients who underwent chloroquine (n = 7) or hydroxychloroquine (n = 480) treatment during their hospital stay.

### 2.2. Data Collection

For each participant, we collected sociodemographic data (age, sex, ethnicity, and living arrangements) and pre-COVID-19 mobility level (classified as able to walk autonomously/with a cane, move around with a walker/wheelchair, and assisted for moving in a wheelchair/bedridden). Concerning chronic diseases, we derived from medical records the presence of arterial hypertension, cardiovascular diseases (CVD, including cardiomyopathy, cardiac failure, or atrial fibrillation), cerebrovascular diseases, diabetes, depressive and cognitive disorders, osteoarticular diseases, chronic obstructive pulmonary disease, chronic kidney disease, chronic liver disease, obesity, and poor nutrition. COVID-19 severity at ward admission was classified based on the indications of the World Health Organization (WHO) [19] as mild (WHO Classes 1, 2 and 3, i.e., no oxygen therapy required), moderate (WHO class 4, i.e., low-flow oxygen requirements), severe (WHO classes 5 and 6, i.e., high-flow oxygen requirement or non-invasive ventilation), and very severe disease (WHO class 7, i.e., need for invasive mechanical ventilation or organ support).

#### 2.2.1. Primary Outcome

The list of medications chronically used by each patient and those administered during the hospitalization were obtained from hospital records and reported through ATC codes. For the present study, the primary outcome was the number of ongoing medications determining possible major interactions with CQ or HCQ. In particular, a list of potentially interacting medications was identified by an expert pharmacologist (AC) according to the current literature and common drug databases, i.e., Micromedex, Codifa, and Medscape (for the complete list, please see Supplementary Table S1).

#### 2.2.2. Secondary Outcomes

As indirect measures of hospitalization-related outcomes, we considered the length of the hospital stay, derived from hospital records, and the patient’s clinical endpoint, classified as discharged, transferred to unspecified or low-intensity care setting, presenting serious adverse events or transferred to intensive care unit, or died.

### 2.3. Statistical Analysis

Continuous variables were expressed as mean and standard deviation (SD), or median and interquartile range (IQR), as appropriate, while categorical variables were expressed as count and percentages. In light of the small number of participants taking CQ and the similarities between CQ and HCQ, we considered together the possible interactions between CQ or HCQ and other medications. The characteristics of patients according to the presence of interactions were compared through an ANOVA or a Kruskal–Wallis test for continuous variables, while the Chi-squared or Fisher tests were used for the categorical variables, as appropriate. The length of stay of patients who did not present any drug interaction with CQ or HCQ vs. those with at least one interaction was compared with the Mann–Whitney test.

Differences in the frequency of clinical outcomes between patients with vs. without drug interactions with chloroquine and hydroxychloroquine were examined using the Chi-squared test. As a sensitivity analysis, we verify whether individuals with drug–drug interactions differed from their counterparts with no interactions by the length of hospital stay or hospitalization outcomes after stratifying the sample by the presence of chronic kidney disease, since both CQ and HCQ undergo renal elimination. Moreover, analyses were performed after excluding individuals who were treated with CQ. Statistical analyses were performed using SPSS version 25.0 statistical packages.

## 3. Results

The mean age of the 487 participants was 77.1 (SD 9.2) years, and 47.8% were females. The main characteristics of the sample are reported in Table 1. As shown, 12.3% lived in a nursing home, and about 30% had low mobility. The most common chronic diseases were CVD, arterial hypertension, osteoarticular diseases, and diabetes. Regarding COVID-19 severity at ward admission, 45% of patients had moderate disease status upon admission, while 25.2% had severe or very severe disease with high oxygen therapy requirements.

**Figure 1:**
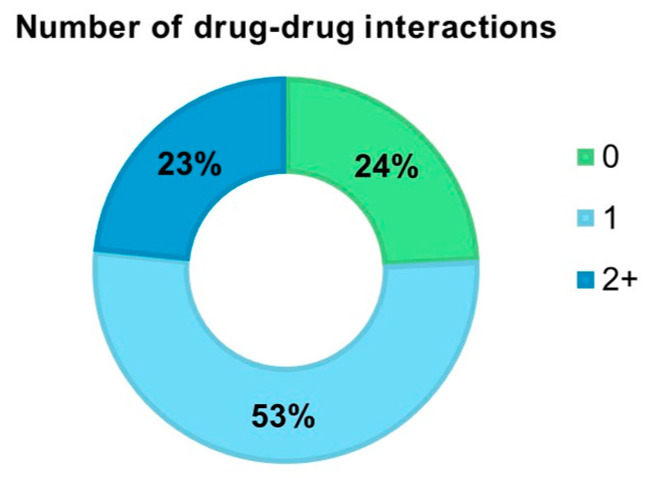
Frequency of COVID-19 patients with major drug–drug interactions with chloroquine or hydroxychloroquine.

Considering the presence of interactions between CQ or HCQ and other drugs, we found that 118 (24.2%) COVID-19 patients had no interactions, 255 (52.4%) presented one interaction, and 114 (23.4%) had two or more drug–drug interactions (Figure 1). Among the drugs potentially interacting with CQ or HCQ in our sample, the most frequent ones were lopinavir/ritonavir (50.4%), azithromycin (47.2%), tocilizumab (15.4%), levofloxacin (8.7%), and clarithromycin (6.0%) (Supplementary Table S2).

When comparing patients according to the number of drug interactions with CQ or HCQ, we found that those with at least two interactions were more likely to be younger, males, to have better mobility and cognitive levels, and to live alone at home. However, they presented with a greater COVID-19 severity at ward admission. Individuals who did not take interacting drugs were more likely to be older, to have lower mobility, and to live in a nursing home. Compared with the other groups, they had the highest number of chronic diseases, especially osteoarticular diseases and cerebrovascular and cognitive disorders, and most of them (82.6%) had mild or moderate COVID-19 (Table 1).

After excluding those who died during the hospital stay (n = 95), we found that the length of hospitalization between patients with no vs. at least one drug interaction with CQ or HCQ did not significantly differ (median 18 [IQR: 10.3–29.8] vs. 18 [IQR: 10–31] days, *p* = 0.98). There were no statistically significant differences, even when considering patients’ clinical outcomes (Table 2), either on the frequency of transfer to low-intensity care settings (27.6% vs. 19.7%, *p* = 0.07, in patients with no vs. at least one drug interaction), ICU transfer or serious adverse events (0% vs. 1.4%, *p* = 0.34), or death (18.1% vs. 20.2%, *p* = 0.62). No substantial differences in these results were observed after excluding individuals treated with CQ (Supplementary Table S3) or when stratifying the sample by the presence of chronic kidney disease.

## 4. Discussion

In older patients hospitalized for COVID-19 during the first two pandemic waves, we found that more than three out of four patients had at least one potential major drug–drug interaction with the ongoing CQ or HCQ therapy. In almost one-quarter of cases, the interactions were two or more and involved mostly other drugs administered during the hospitalization for SARS-CoV-2 infection. Nonetheless, drug–drug interactions with CQ or HCQ did not seem to influence hospitalization length and outcomes. This result suggests that the clinical relevance of these interactions was generally limited, probably because of the relatively low dosage and short treatment duration [20]. Indeed, serious interactions have been shown to occur more frequently in cases of extended treatment with CQ/HCQ [21].

Previous studies evaluated the frequency of drug–drug interactions with off-label medications for COVID-19 in real-world settings, observing results in line with our findings. One work on patients treated with ritonavir/lopinavir found that 78% were taking at least one other potentially interacting drug [22]. In addition, an Italian study on 502 COVID-19 patients, including 320 treated with HCQ, found that 68% had at least one drug–drug interaction and 55% had potentially severe drug–drug interactions. In that study, HCQ was one of the drugs more frequently involved and drove 40% of class D interactions [23]. Similar estimates came from other non-European studies involving smaller samples, which reported that HCQ was among the most frequently interacting drugs in COVID-19 patients [24,25].

Concerning the possible consequences of drug–drug interactions, unlike our results, one previous study observed that those presenting drug interactions with medications against COVID-19 had higher mortality than their counterparts. Still, the study sample, in that case, was not large enough to draw ultimate conclusions [22].

Even irrespective of the results of the main trials testing the effectiveness of HCQ and demonstrating no improvements in COVID-19-related mortality [26,27], regulatory agencies issued updates on HCQ’s efficacy and safety profile in these patients. In particular, the European Medicines Agency (EMA) drew attention to the risks of adverse reactions, sometimes serious, related to CQ or HCQ use. Since administering higher doses of these drugs may further increase the risk of heart rhythm alterations [28], the EMA invited prescribers to monitor HCQ-treated patients carefully. Similarly, the US Food and Drug Administration stressed the need for the monitoring of COVID-19 patients during HCQ treatment, especially in light of safety issues related to severe heart rhythm problems (including tachycardia, atrial fibrillation, and fatal cases of torsade de pointes), often in combination with azithromycin and other medicines that prolong the QT interval [29,30,31]. The latter medications include, for example, some cardiovascular drugs and antipsychotics, which are widely used in geriatric patients [14]. Accordingly, the most common drugs interacting with CQ or HCQ were antibiotics, antivirals, or other medications used off-label for SARS-CoV-2 infection. Among chronic treatments, instead, amiodarone, quetiapine, and trazodone were the most frequent causes of drug–drug interactions with CQ or HCQ. Thus, although HCQ–CQ use is not associated with an increased risk of major cardiac events in randomized trials and drug interactions are not clinically relevant by definition, the current sharp and weakly justified rise in HCQ–CQ prescriptions could pose some clinical challenges [32].

It should be noted that HCQ is 2–3 times less toxic than CQ in experimental in vivo models [33]. Specifically, hepatic, renal, and cardiac adverse effects were milder in rats treated with HCQ than those treated with CQ. HCQ results in a similar, albeit slightly milder, toxicity profile in humans, with lower retinal toxicity than CQ [34]. However, the current recommendation is to use these drugs with caution in patients with a history of liver, neurological, or hematological diseases.

The strengths of this study include the large set of variables collected for a population of COVID-19 patients and the relatively large sample of older individuals treated with HCQ compared with previous study populations. Conversely, the limited resources and personnel to conduct research during the first pandemic waves did not make it possible to collect information on electrocardiographic parameters and other adverse effects caused by the drug–drug interaction. Moreover, for retrospective data collection, some information could not be drawn from medical and hospital records, resulting in missing values (e.g., smoking habits). Finally, we could not explore whether the potential risk of the interactions would be manageable by dose adjustment or other modifying factors [35]. These issues should be evaluated by future studies. However, the availability of data on the clinical outcomes of the hospitalization allowed us to indirectly analyze the possible impact of drug–drug interactions with HCQ or CQ in terms of length of hospital stay and mortality.

In conclusion, in light of the HCQ/CQ experience during the COVID-19 pandemic, our study underlines that, besides verifying the risk–benefit ratio of each new therapy, attention should be paid to assessing possible interactions between newly introduced and other ongoing drugs. This issue concerns especially older patients who are more likely to present multiple chronic diseases and, therefore, pharmacological treatments.

## Figures and Tables

**Table 1:** Characteristics of the study population according to the number of interactions between ongoing therapies and chloroquine/hydroxychloroquine.

	All (n = 487)	Number of Interactions with Chloroquine/Hydroxychloroquine			*p*-Value
		0 (n = 118)	1 (n = 255)	2+ (n = 114)	
Age (years)	77.1 (9.2)	79.2 (8.2)	77.70 (9.3)	73.57 (8.8)	<0.001
Sex (female)	233 (47.8)	66 (55.9)	126 (49.4)	41 (36.0)	0.007
Ethnicity					0.288
*Asian*	1 (0.2)	1 (0.9)	0 (0.0)	0 (0.0)	
*African American*	2 (0.4)	1 (0.9)	1 (0.4)	0 (0.0)	
*Caucasian*	453 (99.1)	110 (98.2)	237 (99.6)	106 (99.1)	
*Other*	1 (0.2)	0 (0.0)	0 (0.0)	1 (0.9)	
Living arrangement					<0.001
*At home, alone*	301 (61.8)	59 (50.0)	155 (60.8)	87 (76.3)	
*At home, assisted*	83 (17.0)	25 (21.2)	46 (18.0)	12 (10.5)	
*Institutionalized*	60 (12.3)	22 (18.6)	35 (13.7)	3 (2.6)	
Smoking habits					0.090
*Never*	170 (34.9)	45 (38.1)	85 (33.3)	40 (35.1)	
*Former*	78 (16.0)	15 (12.7)	36 (14.1)	27 (23.7)	
*Current*	13 (2.7)	2 (1.7)	6 (2.4)	5 (4.4)	
Mobility level					0.001
*Walks alone/with cane*	333 (68.4)	64 (54.2)	177 (69.4)	92 (80.7)	
*Moves with a walker or a wheelchair*	71 (14.6)	26 (22.0)	37 (14.5)	8 (7.0)	
*Moves with a wheelchair assisted/bedridden*	64 (13.1)	24 (20.3)	30 (11.8)	10 (8.8)	
Chronic conditions					
*Diabetes mellitus*	146 (30.0)	32 (27.1)	72 (28.2)	42 (36.8)	0.184
*Liver diseases*	13 (2.7)	4 (3.4)	6 (2.4)	3 (2.6)	0.846
*Osteoarticular diseases*	150 (30.8)	48 (40.7)	75 (29.4)	27 (23.7)	0.015
*Hypertension*	377 (77.4)	96 (81.4)	197 (77.3)	84 (73.7)	0.375
*CVD*	319 (65.5)	81 (68.6)	175 (68.6)	63 (55.3)	0.032
*Ischemic cerebrovascular disease*	54 (11.1)	24 (20.3)	21 (8.2)	9 (7.9)	0.001
*Chronic respiratory diseases*	79 (16.2)	20 (16.9)	38 (14.9)	21 (18.4)	0.678
*CKD*	66 (13.6)	17 (14.4)	37 (14.5)	12 (10.5)	0.559
*Depressive disorders*	93 (19.1)	30 (25.4)	45 (17.6)	18 (15.8)	0.122
*Cognitive disorders*	61 (12.5)	25 (21.2)	31 (12.2)	5 (4.4)	0.001
*Malnutrition*	53 (10.9)	11 (9.3)	32 (12.5)	10 (8.8)	0.461
*Obesity*	102 (20.9)	27 (22.9)	49 (19.2)	26 (22.8)	0.617
WHO disease status at ward admission					0.006
*Mild*	141 (29.3)	40 (34.8)	72 (28.5)	29 (25.4)	
*Moderate*	218 (45.2)	55 (47.8)	123 (48.6)	40 (35.1)	
*Severe*	106 (22.0)	18 (15.7)	50 (19.8)	38 (33.3)	
*Very severe*	17 (3.5)	2 (1.7)	8 (3.2)	7 (6.1)	

Abbreviations: CVD, cardiovascular diseases; CKD, chronic kidney disease; WHO, World Health Organization. Notes. Missing values in ethnic origin (n = 30), living arrangement (n = 43), smoking habits (n = 226), mobility level (n = 19), WHO status at ward admission (n = 5).

**Table 2:** Clinical outcomes of the study participants based on the presence of drug–drug interactions with chloroquine or hydroxychloroquine.

Clinical Outcome	Number of Drug–Drug Interactions with Chloroquine/Hydroxychloroquine		*p*-Value
	No (n = 116)	At Least One (n = 366)	
Discharged stable/improved	63 (54.3)	215 (58.7)	0.40
Transfer to unspecified or low-intensity care	32 (27.6)	72 (19.7)	0.07
ICU transfer or adverse events	0 (0.0)	5 (1.4)	0.34
Death	21 (18.1)	74 (20.2)	0.62

Notes. n = 5 participants had missing information on clinical outcomes.

## Data Availability

Data can be available upon request to the study coordinators, as indicated in the project website (https://www.sigg.it/studio-gerocovid-observational/ (accessed on 15 May 2024)).

## Supplementary Materials

The following supporting information can be downloaded at: https://www.mdpi.com/article/10.3390/reports7020042/s1, Complete list of the GeroCovid acute ward working group; Table S1: List of drugs determining major drug-drug interactions with hydroxychloroquine or chloroquine; Table S2: Drugs interacting with chloroquine or hydroxychloroquine in the 369 patients with COVID-19 presenting drug-drug interactions; Table S3: Clinical outcomes of the study participants based on the presence of drug-drug interactions with hydroxychloroquine (*n* = 476).
