# Supplementary material for: Drug–Drug Interactions of Hydroxychloroquine and Chloroquine in Older Patients with COVID-19 during the First Pandemic Waves: The GeroCovid Observational Study
Source: Reports (MDPI). 2024 May 23;7(2):42. doi: 10.3390/reports7020042 (PMC12225520; doi:10.3390/reports7020042)
Supplement: Supplementary file 1 [file reports-07-00042-s001.zip › reports-2977649-supplementary.pdf]

SUPPLEMENTARY MATERIAL

Complete list of the GeroCovid acute ward working group (alphabetical order)

Rachele Antognoli (Azienda Ospedaliero Universitaria Pisana), **Raffaele Antonelli Incalzi** (Policlinico Universitario Campus Bio-Medico, Roma), **Maria Paola Antonietti** (Ospedale Regionale di Aosta), **Viviana Bagalà** (Azienda Ospedaliero-Universitaria di Ferrara), **Giulia Bandini** (USL Toscana Centro, Ospedale San Jacopo, Pistoia), **Giuseppe Bellelli** (Ospedale San Gerardo, Monza), **Enrico Benvenuti** (USL Toscana Centro, Ospedale Santa Maria Annunziata, Bagno a Ripoli (FI)), **Marina Bergamin** (Azienda Ospedaliero-Universitaria di Parma), **Marco Bertolotti** (Azienda Ospedaliero-Universitaria di Modena), **Carlo Adriano Biagini** (USL Toscana Centro, Ospedale San Jacopo, Pistoia), **Angelo Bianchetti** (Istituto Clinico Sant'Anna, Brescia), **Alessandra Bianchi** (Spedali Civili, Montichiari (BS)), **Mariangela Bianchi** (Policlinico Sant'Orsola-Malpighi, Bologna), **Silvia Bignamini** (Casa di Cura San Francesco, Bergamo), **Damiano Blandini** (Policlinico Sant'Orsola-Malpighi, Bologna), **Stefano Boffelli** (Fondazione Poliambulanza, Brescia), **Maura Bugada** (Casa di Cura San Francesco, Bergamo), **Valeria Calsolaro** (Azienda Ospedaliero Universitaria Pisana), **Donatella Calvani** (USL Toscana Centro, Presidio Misericordia e Dolce, Prato), **Elisiana Carpagnano** (Ospedale Giovanni XXIII Policlinico di Bari), **Barbara Carrieri** (IRCCS INRCA, Ancona), **Viviana Castaldo** (Presidio Ospedaliero Universitario Santa Maria della Misericordia, Udine), **Alessandro Cavarape** (Presidio Ospedaliero Universitario Santa Maria della Misericordia, Udine), **Ilaria Cazzulani** (Ospedale San Gerardo, Monza), **Carilia Celesti** (Policlinico Universitario Campus Bio-Medico, Roma), **Chiara Ceolin** (Azienda Ospedale Università di Padova), **Maria Giorgia Ceresini** (Azienda Ospedaliero-Universitaria di Ferrara), **Antonio Cherubini** (IRCCS INRCA, Ancona), **Anita Chizzoli** (Istituto Clinico Sant'Anna, Brescia), **Erika Ciarrocchi** (IRCCS INRCA, Ancona), **Paola Cicciomessere** (Azienda Ospedaliero Universitaria di Foggia), **Alessandra Coin** (Azienda Ospedale Università di Padova), **Annalisa Corsi** (USL Toscana Centro, Ospedale San Jacopo, Pistoia), **Carlo Custodero** (Ospedale Giovanni XXIII Policlinico di Bari), **Federica D'Agostino** (Casa Sollievo della Sofferenza, San Giovanni Rotondo (FG)), **Maria Maddalena D'Errico** (Casa Sollievo della Sofferenza, San Giovanni Rotondo (FG)), **Aurelio De Iorio** (Azienda Ospedaliero-Universitaria di Parma), **Alessandro De Marchi** (Policlinico Sant'Orsola-Malpighi, Bologna), **Giovambattista Desideri** (Ospedale di Avezzano (AQ)), **Evelyn Di Matteo** (Policlinico Universitario Campus Bio-Medico, Roma), **Emma Espinosa** (Azienda Ospedali Riuniti Marche Nord, Fano (PU)), **Luigi Esposito** (Casa Sollievo della Sofferenza, San Giovanni Rotondo (FG)), **Chiara Fazio** (Azienda Ospedaliero-Universitaria di Parma), **Chiara Filippini** (Spedali Civili, Montichiari (BS)), **Lucia Fiore** (Casa Sollievo della Sofferenza, San Giovanni Rotondo (FG)), **Caterina Fontana** (Azienda Ospedaliero-Universitaria di Modena), **Lina Forte** (Ospedale di Avezzano (AQ)), **Riccardo Franci Montorzi** (Azienda Ospedaliero Universitaria Careggi, Firenze), **Carlo Fumagalli** (Azienda Ospedaliero Universitaria Careggi, Firenze), **Stefano Fumagalli** (Azienda Ospedaliero Universitaria Careggi, Firenze), **Pietro Gareri** (CDCD Catanzaro Lido, ASP Catanzaro), **Antonella Giordano** (Azienda Ospedaliero Universitaria Careggi, Firenze), **Evelina Giuliani** (USL Toscana Centro, Ospedale Santa Maria Annunziata, Bagno a Ripoli (FI)), **Antonio Greco** (Casa Sollievo della Sofferenza, San Giovanni Rotondo (FG)), **Andrea Herbst** (Azienda Ospedaliero Universitaria Careggi, Firenze), **Giuseppe Ielo** (Azienda Ospedaliero-Universitaria di Parma), **Antonella La Marca** (Casa Sollievo della Sofferenza, San Giovanni Rotondo (FG)), **Umberto La Porta** (Azienda Ospedaliero-Universitaria di Parma), **Ilaria Lazzari** (Policlinico Sant'Orsola-Malpighi, Bologna), **Diana Lelli** (Policlinico Universitario Campus Bio-Medico, Roma), **Yari Longobucco** (Azienda Ospedaliero-Universitaria di Parma), **Flaminia Lucchini** (Azienda Ospedaliero

Universitaria Careggi, Firenze), **Daniela Lucente** (Spedali Civili, Montichiari (BS)), **Lorenzo Maestri** (Policlinico Sant'Orsola-Malpighi, Bologna), **Marcello Maggio** (Azienda Ospedaliero-Universitaria di Parma), **Paola Mainquà** (Azienda Ospedali Riuniti Marche Nord, Fano (PU)), **Alessandra Marengoni** (Spedali Civili, Montichiari (BS)), **Benedetta Martin** (Ospedale di Avezzano (AQ)), **Valentina Massa** (Casa Sollievo della Sofferenza, San Giovanni Rotondo (FG)), **Liliana Mazza** (Policlinico Sant'Orsola-Malpighi, Bologna), **Carmela Mazzoccoli** (Ospedale Giovanni XXIII Policlinico di Bari), **Fabio Monzani** (Azienda Ospedaliero Universitaria Pisana), **Enrico Mossello** (Azienda Ospedaliero Universitaria Careggi, Firenze), **Federica Morellini** (Azienda Ospedaliero-Universitaria di Modena), **Chiara Mussi** (Azienda Ospedaliero-Universitaria di Modena), **Chukwuma Okoye** (Azienda Ospedaliero Universitaria Pisana), **Giuseppe Orio** (Policlinico Sant'Orsola-Malpighi, Bologna), **Annalisa Paglia** (Azienda Ospedaliero Universitaria di Foggia), **Giulia Pelagalli** (Azienda Ospedaliero Universitaria Careggi, Firenze), **Laura Pelizzoni** (Policlinico Sant'Orsola-Malpighi, Bologna), **Alessandro Picci** (Presidio Ospedaliero Universitario Santa Maria della Misericordia, Udine), **Anette Hylen Ranhoff** (University of Bergen, Norway), **Francesca Remelli** (Azienda Ospedaliero-Universitaria di Ferrara), **Onofrio Resta** (Ospedale Giovanni XXIII Policlinico di Bari), **Antonella Riccardi** (Policlinico Sant'Orsola-Malpighi, Bologna), **Daniela Rinaldi** (Ospedale di Comunità (Camposampiero), Distretto Alta Padovana, ULSS 6 Euganea, Padova), **Renzo Rozzini** (Fondazione Poliambulanza, Brescia), **Carlo Sabbà** (Ospedale Giovanni XXIII Policlinico di Bari), **Leonardo Sacco** (Casa Sollievo della Sofferenza, San Giovanni Rotondo (FG)), **Mariateresa Santoliquido** (Casa Sollievo della Sofferenza, San Giovanni Rotondo (FG)), **Mariella Savino** (Casa Sollievo della Sofferenza, San Giovanni Rotondo (FG)), **Francesco Scarso** (Azienda Ospedaliero-Universitaria Sant'Andrea, Roma), **Giuseppe Sergi** (Azienda Ospedale Università di Padova), **Gaetano Serviddio** (Azienda Ospedaliero Universitaria di Foggia), **Chiara Sidoli** (Ospedale San Gerardo, Monza), **Vincenzo Solfrizzi** (Ospedale Giovanni XXIII Policlinico di Bari), **Benedetta Soli** (Azienda Ospedaliero-Universitaria di Modena), **Laura Tafaro** (Azienda Ospedaliero-Universitaria Sant'Andrea, Roma), **Andrea Tedde** (Azienda Ospedaliero-Universitaria di Modena), **Giuseppe Dario Testa** (USL Toscana Centro, Ospedale San Jacopo, Pistoia), **Maria Giulia Tinti** (Casa Sollievo della Sofferenza, San Giovanni Rotondo (FG)), **Francesco Tonarelli** (USL Toscana Centro, Presidio Misericordia e Dolce, Prato), **Elisabetta Tonon** (USL Toscana Centro, Ospedale San Jacopo, Pistoia), **Caterina Trevisan** (Ospedale di Comunità (Camposampiero), Distretto Alta Padovana, ULSS 6 Euganea, Padova; Azienda Ospedale Università di Padova), **Aurora Vitali** (Azienda Ospedaliero-Universitaria di Ferrara), **Stefano Volpato** (Azienda Ospedaliero-Universitaria di Ferrara), **Francesca Zoccarato** (Azienda Ospedale Università di Padova), **Sonia Zotti** (Policlinico Universitario Campus Bio-Medico, Roma).

Supplementary Table S1. List of drugs determining major drug-drug interactions with hydroxychloroquine or chloroquine.

**Severe drug interactions with hydroxychloroquine**			
**Adalimumab**<br>**Alfuzosin**<br>**Amiodarone**<br>**Amisulpride**<br>**Amitriptyline**<br>**Amoxapine**<br>**Anagrelide**<br>**Anakinra**<br>**Apomorphine**<br>**Aripiprazole**<br>**Arsenic Trioxide**<br>**Artemether**<br>**Artemether/ Lumefantrine**<br>**Asenapine**<br>**Atomoxetine**<br>**Axicabtagene Ciloleucel**<br>**Azathioprine**<br>**Azithromycin**<br>**Basiliximab**<br>**BCG Vaccine Live**<br>**Bedaquiline**<br>**Brexucabtagene Autoleucel**<br>**Buprenorphine**<br>**Canakinumab**<br>**Ceritinib**<br>**Chlorpromazine**<br>**Ciltacabtagene Autoleucel**<br>**Ciprofloxacin**<br>**Citalopram**<br>**Clarithromycin**<br>**Clofazimine**<br>**Clomipramine**<br>**Clozapine**<br>**Crizotinib**<br>**Dapsone Topical**<br>**Dasatinib**<br>**Degarelix**<br>**Desflurane**	Desipramine Deutetrabenazine<br>Digoxin<br>Dolasetron<br>Donepezil<br>Doxepin<br>Dronedarone<br>Droperidol<br>Efavirenz<br>Eliglustat<br>Encorafenib<br>Entrectinib<br>Eribulin<br>Erythromycin Base<br>Erythromycin Ethylsuccinate<br>Erythromycin Lactobionate<br>Erythromycin Stearate<br>Escitalopram<br>Etanercept<br>Everolimus<br>Fexinidazole<br>Fingolimod<br>Flecainide<br>Fluconazole<br>Fluoxetine<br>Fluphenazine<br>Formoterol<br>Foscarnet<br>Gemtuzumab<br>Gilteritinib<br>Glasdegib<br>Glatiramer<br>Golimumab<br>Granisetron<br>Haloperidol<br>Hydroxyzine<br>Ibutilide<br>Idecabtagene Vicleucel<br>Indacaterol, Inhaled<br>Indapamide	Infliximab<br>Inotuzumab<br>Isoflurane<br>Isradipine<br>Itraconazole<br>Lapatinib<br>Leflunomide<br>Levofloxacin<br>Lisocabtagene Maraleucel<br>Lithium<br>Lofexidine<br>Lopinavir<br>Maprotiline<br>Methadone<br>Mifepristone<br>Mirtazapine<br>Mobocertinib<br>Moxifloxacin<br>Muromonab Cd3<br>Mycophenolate<br>Nilotinib<br>Nortriptyline<br>Octreotide<br>Ofloxacin<br>Olanzapine<br>Ondansetron<br>Osimertinib<br>Oxaliplatin<br>Paliperidone<br>Panobinostat<br>Pasireotide<br>Pazopanib<br>Pentamidine<br>Perphenazine<br>Pimavanserin<br>Pitolisant<br>Posaconazole<br>Procainamide<br>Propafenone<br>Protriptyline Quetiapine	Quinidine<br>Quinine<br>Ranolazine<br>Remdesivir<br>Ribociclib<br>Rilonacept<br>Rilpivirine<br>Risperidone<br>Ritonavir<br>Romidepsin<br>Salbutamolo<br>Saquinavir<br>Sertraline<br>Sevoflurane<br>Siponimod<br>Sirolimus<br>Solifenacin<br>Sorafenib<br>Sotalol<br>Sunitinib<br>Tacrolimus<br>Telavancin<br>Temsirolimus<br>Tetrabenazine<br>Thioridazine<br>Thiothixene<br>Tisagenlecleucel<br>Tocilizumab<br>Tofacitinib<br>Tongkat Ali<br>Toremifene<br>Trimipramine<br>Ustekinumab<br>Vandetanib<br>Vardenafil<br>Vemurafenib<br>Vilanterol/<br>Fluticasone<br>Furoate Inhaled<br>Voriconazole<br>Vorinostat<br>Ziprasidone
**Severe drug interactions with chloroquine**			
Abametapir<br>Amiodarone<br>Amisulpride<br>Amlodipine<br>Anagrelide<br>Apalutamide	Disopyramide<br>Dofetilidedonepezil<br>Doxepin<br>Dronedarone<br>Efavirenz<br>Eliglustat	Itraconazole Ivosidenib<br>Lefamulin<br>Levalbuterol<br>Lithium<br>Lonafarnib<br>Loperamide	Primaquine<br>Procainamide Quinidine<br>Quinine<br>Remdesivir<br>Ribociclib<br>Salmeterol

Aripiprazole	Entrectinib	Lopinavir	Saquinavir
Arsenictrioxide	Eribulin	Lumefantrine	Selpercatinib
Atomoxetine	Fexinidazole	Methadone	Sevoflurane
Bedaquiline	Fingolimod	Midostaurin	Siponimod
Buprenorphine	Gilteritinib	Mirtazapine	Thioridazine
Ceritinib	Givosiran	Olodaterolinhaled	Trazodone
Chlorpromazine	Glasdegib	Oxaliplatin	Triclabendazole
Cimetidine	Granisetron	Ozanimod	Tucatinib
Cisapride	Hydroxyzine	Panobinostat	Vandetanib
Clarithromycin	Idelalisib	Pimozide	Voclosporin
Conivaptan	Influenza virus vaccine tri-	Pitolisant	Voriconazole
Dacomitinib	valent, Adjuvanted	Ponesimod	Voxelotor
Dapsonetopical	Inotuzumab	Posaconazole	Ziprasidone
Desflurane	Isoflurane		

Supplementary Table S2. Drugs interacting with chloroquine or hydroxychloroquine in the 369 patients with COVID-19 presenting drug-drug interactions.

	**Frequency of major drug-drug interactions with chloroquine/hydroxychloroquine (n=369)**
Drugs used for acute diseases	
Ciprofloxacin	1 (0.3)
Clarithromycin	22 (6.0)
Tocilizumab	57 (15.4)
Azithromycin	174 (47.2)
Fluconazole	4 (1.1)
Levofloxacin	32 (8.7)
Lopinavir/Ritonavir	186 (50.4)
Drugs chronically used	
Amiodarone	2 (0.5)
Anakinra	1 (0.3)
Dronedarone	1 (0.3)
Escitalopram	1 (0.3)
Haloperidol	7 (1.9)
Ondansetron	1 (0.3)
Olanzapine	1 (0.3)
Quetiapine	5 (1.4)
Salbutamol	2 (0.5)
Sertraline	1 (0.3)
Salmeterol/Fluticasone	1 (0.3)
Trazodone	9 (2.4)
Amlodipine	12 (3.3)

Abbreviations: HCQ, hydroxychloroquine; CQ, chloroquine.

Supplementary Table S3. Clinical outcomes of the study participants based on the presence of drug-drug interactions with hydroxychloroquine (n=476).

Clinical outcome	N. drug-drug interactions with hydroxychloroquine		p-value
	No (n=114)	At least one (n=362)	
**Discharged stable/improved**	63 (55.3)	213 (58.8)	0.50
Transfer to Unspecified or Low-Intensity Care	31 (27.2)	71 (19.6)	0.09
ICU Transfer or Adverse Events	0 (0.0)	5 (1.4)	0.21
Death	20 (17.5)	73 (20.2)	0.54

Notes. N=4 participants had missing information on clinical outcomes.
